# Mining hidden knowledge for drug safety assessment: topic modeling of LiverTox as a case study

**DOI:** 10.1186/1471-2105-15-S17-S6

**Published:** 2014-12-16

**Authors:** Ke Yu, Jie Zhang, Minjun Chen, Xiaowei Xu, Ayako Suzuki, Katarina Ilic, Weida Tong

**Affiliations:** 1 Division of Bioinformatics and Biostatistics, National Center for Toxicological Research, US Food and Drug Administration, 3900 NCTR Road, Jefferson, AR 72079, USA; 2 Department of Information Science, University of Arkansas at Little Rock, 2801 S. University Ave., Little Rock, AR 72204, USA; 3 Division of Gastroenterology, University of Arkansas for Medical Sciences, 4301 W. Markham St., Little Rock, AR 72205, USA; 4 Department of Pharmacology, Faculty of Pharmacy, University of Belgrade, 450 Vojvode Stepe, Belgrade 11221, Serbia

## Abstract

**Background:**

Given the significant impact on public health and drug development, drug safety has been a focal point and research emphasis across multiple disciplines in addition to scientific investigation, including consumer advocates, drug developers and regulators. Such a concern and effort has led numerous databases with drug safety information available in the public domain and the majority of them contain substantial textual data. Text mining offers an opportunity to leverage the hidden knowledge within these textual data for the enhanced understanding of drug safety and thus improving public health.

**Methods:**

In this proof-of-concept study, topic modeling, an unsupervised text mining approach, was performed on the LiverTox database developed by National Institutes of Health (NIH). The LiverTox structured one document per drug that contains multiple sections summarizing clinical information on drug-induced liver injury (DILI). We hypothesized that these documents might contain specific textual patterns that could be used to address key DILI issues. We placed the study on drug-induced acute liver failure (ALF) which was a severe form of DILI with limited treatment options.

**Results:**

After topic modeling of the "Hepatotoxicity" sections of the LiverTox across 478 drug documents, we identified a hidden topic relevant to Hy's law that was a widely-accepted rule incriminating drugs with high risk of causing ALF in humans. Using this topic, a total of 127 drugs were further implicated, 77 of which had clear ALF relevant terms in the "Outcome and management" sections of the LiverTox. For the rest of 50 drugs, evidence supporting risk of ALF was found for 42 drugs from other public databases.

**Conclusion:**

In this case study, the knowledge buried in the textual data was extracted for identification of drugs with potential of causing ALF by applying topic modeling to the LiverTox database. The knowledge further guided identification of drugs with the similar potential and most of them could be verified and confirmed. This study highlights the utility of topic modeling to leverage information within textual drug safety databases, which provides new opportunities in the big data era to assess drug safety.

## Background

In recent years, numerous drug safety databases have been made publicly available, e.g., LiverTox http://livertox.nlm.nih.gov/[1], SIDER http://sideeffects.embl.de/[2], TOXNET http://toxnet.nlm.nih.gov/, the US Food and Drug Administration (FDA) Adverse Event Reporting System (FAERS), and PubMed http://www.ncbi.nlm.nih.gov/pubmed. These databases contribute significantly to the research community, facilitating the enhanced understanding of drug safety issues [3]. Mining large-scale drug safety data is a promising venue for drug regulation [4]. Some databases integrated the safety data from various sources with free text format, for which text mining would be effective to leverage the textual information to gain knowledge of drug safety, and thus address critical safety issues that are difficult to be approached by using other databases.

Topic modeling is a widely used text mining approach for analysis of large volumes of unlabeled documents in order to discover hidden textual patterns [5]. Previous studies in our group demonstrated that topic modeling could be effectively used for the analysis of adverse events for drug safety assessment from the FDA-approved drug labels [6], and for the identification of opportunities for drug repositioning [7]. The National Institutes of Health (NIH) LiverTox database provides comprehensive clinical information for drug-induced liver injury (DILI) which is summarized with a free-text format in several sections.

In this study, we extended our text mining effort with topic modeling to the LiverTox database to ask the question of whether additional knowledge beyond what had been described in the documents could be extracted to guide an enhanced DILI assessment. We placed our emphasis on drug-induced acute liver failure (ALF) which was a severe form of DILI with limited treatment options thus with significant public health impact. With topic modeling, we successfully identified a topic incriminating a drug's liability to cause ALF based on the text in the "Hepatotoxicity" sections of the LiverTox. The identified topic further guided identification of other drugs with the similar liability and, importantly, most of them could be verified and confirmed with additional data. This proof-of-concept study demonstrated the potential utility of topic modeling to the existing text documents in the public domain to gain knowledge as predictive means for the enhanced assessment of drug safety.

## Methods

### LiverTox database

The NIH LiverTox database is developed by Liver Disease Research Branch of National Institute of Diabetes and Digestive and Kidney Diseases (NIDDK) and National Library of Medicine (NLM) to promote the basic and clinical research on DILI [1]. It is a free on-line source of textual documents on DILI summarized from various databases, scientific literature, and interpretations of the curators. The LiverTox contains a set of documents (one document per drug) and each document contains multiple sections. Each section provides different set of DILI information, including introduction, background, hepatotoxicity, mechanism of injury, outcome and management, and others (e.g., case report, product information, chemical structure, and references). In this study, only the "Hepatotoxicity" section was used for topic modeling because the "Hepatotoxicity" section mainly contained the DILI-relevant clinical observations. The findings was compared against to the information from other sections such as "Outcome and management" to demonstrate the utility of the method. In case of no clear ALF evidence presented in those sections, the results were compared to the data from other sources. The "Hepatotoxicity" section for each drug, on average, contains 200-400 words that summarize the DILI-relevant information including clinical features, time to onset and recovery, liver enzymes (frequency of elevation, fold change, and serum levels), liver injury pattern, immunoallergic and autoimmune features, and other hepatotoxicity relevant data. A total of 478 documents (i.e., 478 drugs) were used for topic modeling.

### Topic modeling

Latent Dirichlet allocation (LDA), one of the most popular topic modeling approach [5,8-10], was applied to explore the LiverTox database. We used LDA in Mallet, a Java-based package, for topic modeling [11]. Number of topics to optimally represent the content of all documents is a key parameter in a topic model. The optimal number of topics can be determined by fitting models with different number of topics to the data. The model fitness can be estimated by the likelihood of the data given a topic model [10]. To obtain the sparse topic and word distributions, the Dirichlet hyperparameters alpha (α) and beta (β) were defined as 0.1 and 0.01, respectively. Before topic modeling, the English stop-words and numerical digits are removed. In addition, three words (i.e., *liver*, *injury*, and *elevation*) presented in more than 80% of documents are also removed as the words with high frequency across the documents will not provide the discriminative information for topics. After that, words in each document are tokenized and then put into LDA to train a topic model. The model yields two probability distributions, one gives a probability value (*θ*) for each topic to determine its relevance to each document and the other assigns a probability value to each word for its relevance to the topic.

### Identification of ALF-Topic

As listed in Table 1, 26 drugs known to cause ALF are selected and used to identify a topic most relevant to ALF in the topic model. There are 23 drugs annotated by Suzuki *et al*. with a justified causality assessment from the ALF survey conducted in the United States [12]. Another 3 drugs (i.e., benzbromarone, tolcapone, and troglitazone) are withdrawn from the market due to the drug-induced ALF.

**Table 1:** Summary of topic model for the 26 known ALF drugs in LiverTox database.

Known ALF drugs	1^st ^topic		2^nd ^topic		3^rd ^topic		4^th ^topic	
	
	Topic ID	**Prob**.	Topic ID	**Prob**.	Topic ID	**Prob**.	Topic ID	**Prob**.
Acetaminophen	**37**	4.7E-01	29	3.6E-01	4	1.4E-01	6	1.6E-02
Amoxicillin/clavulanate	**37**	6.3E-01	4	1.9E-01	15	1.2E-01	20	6.1E-02
Atorvastatin	31	8.3E-01	39	1.7E-01	40	3.3E-05	38	3.3E-05
Benzbromarone*	28	4.4E-01	**37**	4.2E-01	11	1.4E-01	40	3.8E-05
Carbamazepine	3	4.6E-01	35	3.5E-01	38	1.9E-01	40	6.7E-05
Ciprofloxacin	15	7.7E-01	14	2.3E-01	40	2.8E-05	39	2.8E-05
Cyclophosphamide	31	6.4E-01	19	2.9E-01	40	6.2E-02	39	5.2E-05
Dapsone	7	8.0E-01	31	2.0E-01	40	3.3E-05	39	3.3E-05
Diclofenac	**37**	8.0E-01	20	1.4E-01	23	3.3E-02	22	2.2E-02
Disulfiram	**37**	3.6E-01	38	2.6E-01	28	2.2E-01	7	1.6E-01
Doxycycline	**37**	5.9E-01	17	3.3E-01	22	7.2E-02	40	3.6E-05
Ethambutol	28	3.9E-01	10	3.5E-01	7	1.7E-01	24	8.7E-02
Halothane	23	7.1E-01	**37**	2.8E-01	16	6.5E-03	40	1.6E-05
Ibuprofen	31	3.2E-01	21	2.9E-01	**37**	2.0E-01	16	1.3E-01
Isoniazid	**37**	6.2E-01	23	1.2E-01	10	9.5E-02	16	6.7E-02
Methyldopa	**37**	7.9E-01	20	2.1E-01	40	5.2E-05	39	5.2E-05
Naproxen	**37**	6.2E-01	30	1.8E-01	20	1.6E-01	2	3.6E-02
Nefazodone	**37**	4.2E-01	26	2.7E-01	24	1.8E-01	28	1.3E-01
Nitrofurantoin	**37**	1.0E+00	40	4.6E-05	39	4.6E-05	38	4.6E-05
Phenytoin	7	3.0E-01	**37**	2.5E-01	3	2.1E-01	35	1.6E-01
Simvastatin	31	4.9E-01	**37**	4.4E-01	34	6.1E-02	26	1.0E-02
Sulfamethoxazole/ trimethoprim	7	8.3E-01	28	1.7E-01	40	3.5E-05	39	3.5E-05
Sulfasalazine	7	6.8E-01	**37**	3.2E-01	40	3.2E-05	39	3.2E-05
Tolcapone*	**37**	6.4E-01	25	2.7E-01	3	8.7E-02	40	3.1E-05
Troglitazone*	**37**	4.6E-01	31	4.2E-01	1	1.1E-01	40	2.2E-05
Valproate	35	4.2E-01	38	3.1E-01	26	2.1E-01	14	5.2E-02

^*^These three drugs are withdrawn from the market due to the drug-induced ALF.

Topic-37 is the ALF-Topic.

Topic ID: serial number of topics in topic model.

Prob.: conditional probability of each topic to a drug.

Topic model ranks topics in accordance to their probability to each drug.

For the 26 known ALF drugs, a mean topic distribution of these drugs is calculated, which leads to the determination of a topic that represents best for these ALF drugs. This so-called ALF-Topic is defined to be topic *j *for which the mean probability value of *θ*_j_ is the greatest among all the topics. In this model, other drugs highly associated with ALF-Topic are expected to be related to ALF.

### Investigation of drugs implicated by ALF-Topic

**Figure 1:**
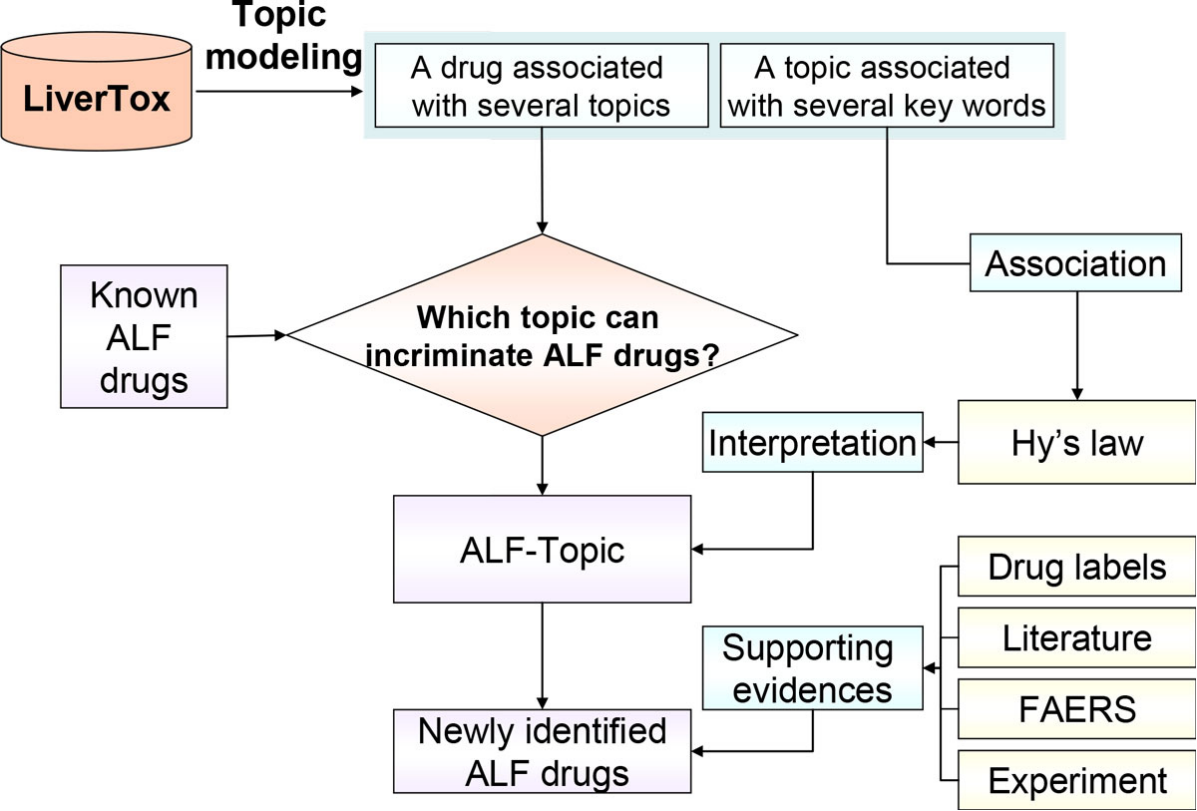
**Workflow of this study**. The 26 known ALF drugs are from the Suzuki's paper [12]. ALF: acute liver failure; FAERS: FDA Adverse Event Reporting System; Hy's law: a well-accepted rule to incriminate ALF [17].

To investigate whether there was any evidence to support the ALF-implicated drugs identified by ALF-Topic, we searched the ALF evidence in their "Outcome and management" sections from the LiverTox database, in the safety sections from the FDA-approved drug labels, in the literature reporting the ALF case reports with the established causality, and in the FAERS with post-marketing ALF case reports from 1969 to 2013. The workflow of this study is depicted in Figure 1.

## Results

### Identification of ALF-Topic

**Figure 2:**
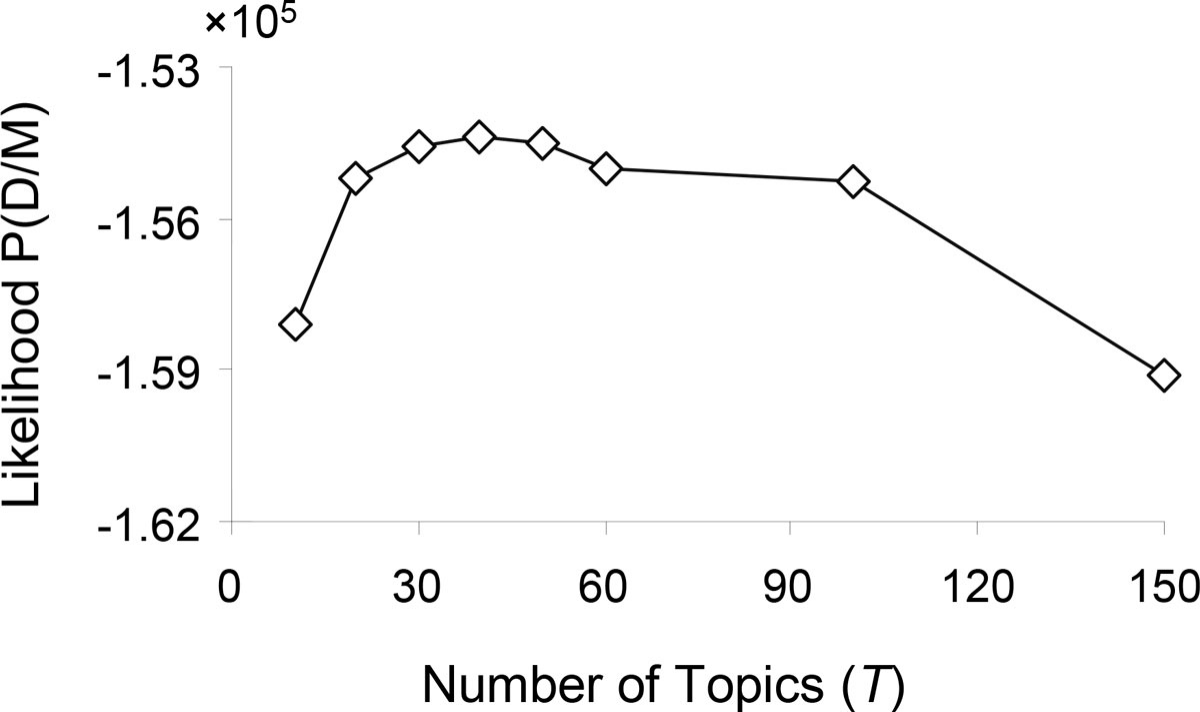
**Log-likelihood of the data (D) given the model (M) with different settings of the number of topics (*T*)**.

**Figure 3:**
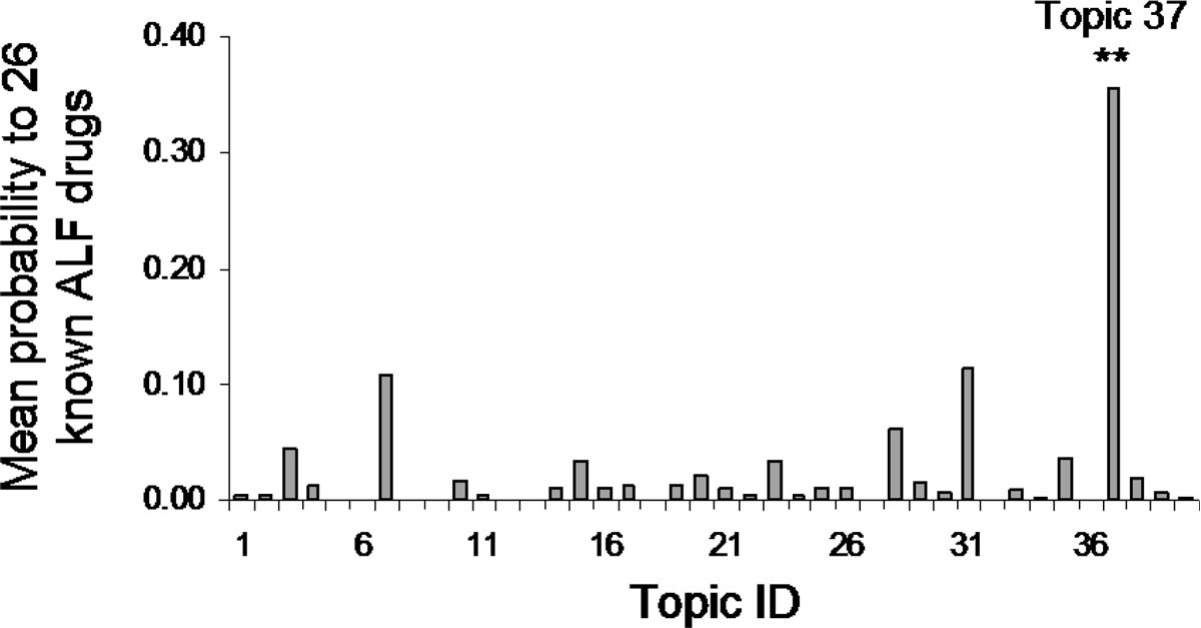
Mean probability values of all 40 topics for the 26 known ALF drugs

The study started with the determination of the optimal number of topics for the LiverTox dataset. Consequently, 40 topics were determined as the highest likelihood of the data given the model with the varying number of topics between 10 and 150 (Figure 2). Then, the mean probability value (*θ*) for each topic was calculated for 26 known ALF drugs. As shown in Figure 3, Topic-37 had significantly higher probability value (0.36) to these drugs compared to the baseline (0.02) from the rest of topics (*p *< 0.01). Therefore, this topic was considered as an ALF recognizing/predicting topic and denoted as ALF-Topic.

The following 15 words were prevalently represented in ALF-Topic: "*case*, *acute*, *hepatic*, *therapy*, *serum*, *pattern*, *week*, *clinical*, *report*, ***jaundice***, ***hepatocellular***, *patient*, *typical*, ***severe***, *aminotransferase*". Three of these words (i.e., *jaundice*, *hepatocellular*, and *severe*) were unique to this topic and were not simultaneously present in first 15 words for the other 39 topics. These specific words might imply a clinical phenomenon likely to indicate the potential of a drug to cause ALF. Thus, ALF-Topic could be applied to identify other ALF-related drugs in this model based on the probability values of this topic to those drugs.

### Application of ALF-Topic

For 12 (12/26; ~46%) known ALF drugs listed in Table 1, ALF-Topic (i.e., Topic-37) was ranked as the first topic, and this proportion was significantly higher compared to that of the other topics (*p *< 0.05). For five other drugs (i.e., Benzbromarone, Halothane, Phenytoin, Simvastatin, and Sulfasalazine), ALF-Topic was ranked as the second, while for Ibuprofen it was in the third place. There were no ALF drugs with ALF-Topic ranked in the fourth place. The results suggested that a drug with ALF-Topic ranked among its first three topics might have a high likelihood to be associated with ALF. This criterion (i.e., ALF-associated drugs would have ALF-Topic ranked among their first three topics in the model) was applied to the rest of drugs in the LiverTox database, and a total of 127 drugs were identified.

### Confirmation of drugs identified by ALF-Topic

Among the identified drugs, 77/127 (60.6%) drugs were described in their "Outcome and management" sections with the ALF-related terms such as "liver failure", "hepatic failure", "liver transplantation", "fatal/death", or "fulminant hepatitis" (Additional file 1: Table S1). The remaining 50/127 (39.4%) ALF-implicated drugs were not mentioned to cause ALF in the LiverTox database (Table 2). We examined the safety sections in the FDA-approved drug labels, and found out that 13/50 (26%) drugs were mentioned to have ALF risk in the Warnings & Precautions, and/or Adverse reactions sections (Table 2). For another 7 drugs (7/50; 14%), there were reports for drug-induced ALF with the established causality in literature [12-15]. For the remaining 30 drugs, we found that 22 (22/50; 44%) drugs had the ALF case reports in the FAERS (Additional file 2: Table S2), which were obtained by searching the FAERS with the Medical Dictionary for Regulatory Activities (MedDRA) preferred terms: "*acute hepatic failure*" and/or "*hepatic failure*". Apart from 4 herbal medicines (i.e, Aloe Vera, Ba Jiao Lian, Chi R Yun, and Shosaikoto/daisaikoto), which were not recorded by the FAERS, no ALF case was reported for the remaining 4 drugs (i.e., Clofibrate, Methocarbamol, Pentamidine, and Reserpine). In summary, among 127 identified drugs, evidence supporting risk of ALF was found for 119 drugs (119/127; 93.7%).

**Table 2:** Summary of 50 drugs implicated by ALF-Topic without apparent ALF evidence in the LiverTox database.

Drug name	1^st ^topic		2^nd ^topic		3^rd ^topic	
	
	Topic ID	**Prob**.	Topic ID	**Prob**.	Topic ID	**Prob**.
Atovaquone^†^	**37**	6.2E-01	14	3.8E-01	40	5.9E-05
Buprenorphine^†^	22	4.1E-01	**37**	3.1E-01	31	2.5E-01
Clarithromycin^†^	**37**	5.3E-01	17	2.6E-01	3	1.8E-01
Diflunisal^†^	**37**	3.6E-01	38	2.6E-01	29	2.2E-01
Flecainide^†^	**37**	4.3E-01	18	2.7E-01	30	2.1E-01
Infliximab^†^	**37**	5.4E-01	14	3.6E-01	39	9.6E-02
Methimazole^†^	**37**	6.1E-01	7	3.9E-01	40	4.4E-05
Minocycline^†^	**37**	8.9E-01	35	1.1E-01	40	6.9E-05
Nabumetone^†^	38	4.2E-01	36	4.2E-01	**37**	1.5E-01
Proguanil^†^	**37**	5.8E-01	14	4.2E-01	40	4.0E-05
Salsalate^†^	38	6.4E-01	**37**	1.9E-01	30	1.7E-01
Ticlopidine^†^	**37**	4.5E-01	31	2.9E-01	7	2.5E-01
Topiramate^†^	35	4.9E-01	**37**	3.5E-01	5	1.7E-01
Chlorzoxazone^*^	**37**	6.4E-01	17	2.2E-01	20	1.0E-01
Cocaine^*^	29	4.7E-01	**37**	2.4E-01	23	1.7E-01
Chlorpromazine^*^	13	7.0E-01	21	1.9E-01	**37**	1.1E-01
Greater celandine^*^	**37**	8.2E-01	20	9.8E-02	34	5.9E-02
Hydralazine^*^	**37**	6.7E-01	17	3.0E-01	35	3.4E-02
Jin bu huan^*^	**37**	5.7E-01	27	2.6E-01	14	1.7E-01
Shouwupian^*^	**37**	9.8E-01	27	2.1E-02	40	5.2E-05
Colchicine^‡§^	33	4.0E-01	**37**	2.5E-01	9	1.9E-01
Fenofibrate^‡§^	**37**	8.7E-01	33	1.3E-01	40	2.7E-05
Mebendazole^‡§^	5	4.6E-01	2	2.5E-01	**37**	2.3E-01
Metformin^‡§^	**37**	6.2E-01	10	3.5E-01	32	2.8E-02
Nifedipine^‡§^	**37**	5.9E-01	14	2.2E-01	21	1.7E-01
Verapamil^‡§^	**37**	5.9E-01	21	3.6E-01	9	5.1E-02
Acebutolol^‡^	**37**	5.4E-01	5	4.6E-01	40	3.6E-05
Adefovir^‡^	26	5.9E-01	**37**	3.1E-01	30	9.6E-02
Alfuzosin^‡^	21	6.0E-01	**37**	2.2E-01	30	1.8E-01
Ceftriaxone^‡^	**37**	5.3E-01	33	3.3E-01	7	8.0E-02
Gold salts^‡^	**37**	7.0E-01	39	1.9E-01	4	1.1E-01
Linezolid^‡^	6	3.9E-01	36	2.6E-01	**37**	2.0E-01
Montelukast^‡^	31	4.3E-01	**37**	3.4E-01	5	2.1E-01
Olanzapine^‡^	**37**	5.9E-01	13	1.8E-01	19	1.7E-01
Orphenadrine^‡^	29	5.8E-01	**37**	3.7E-01	11	5.2E-02
Oxacillin^‡^	**37**	5.8E-01	4	3.7E-01	12	4.5E-02
Phenelzine^‡^	31	6.2E-01	**37**	3.6E-01	1	1.9E-02
Risperidone^‡^	**37**	6.4E-01	13	3.2E-01	18	2.9E-02
Sotalol^‡^	32	4.7E-01	**37**	4.3E-01	7	1.0E-01
Thioridazine^‡^	13	8.4E-01	**37**	1.6E-01	40	4.0E-05
Ticarcillin/clavulanate^‡^	**37**	4.5E-01	4	3.2E-01	3	2.3E-01
Trisalicylate^‡^	38	5.4E-01	**37**	4.6E-01	40	4.1E-05
Clofibrate	**37**	4.0E-01	31	3.4E-01	33	2.6E-01
Methocarbamol	**37**	3.5E-01	25	3.3E-01	38	2.9E-01
Pentamidine	**37**	7.7E-01	29	2.3E-01	40	5.7E-05
Reserpine	**37**	5.3E-01	36	3.2E-01	9	9.3E-02
Aloe Vera	**37**	6.1E-01	9	2.8E-01	36	1.1E-01
Ba Jiao Lian	25	5.9E-01	**37**	4.1E-01	40	6.4E-05
Chi R Yun	25	3.2E-01	**37**	2.7E-01	11	2.4E-01
Shosaikoto/daisaikoto	**37**	7.6E-01	23	2.4E-01	40	3.8E-05

^†^26% (13/50) drugs confirmed by safety concerns about ALF in the FDA-approved drug labels.

^*^14% (7/50) drugs confirmed by the literature with justified ALF causality.

^‡^44% (22/50) drugs confirmed by ALF case reports in the FAERS.

^§^These 6 drugs were predicted as ALF positive drugs by the *in vitro *experiment.

Topic-37 is the ALF-Topic.

Topic ID: serial number of topics in topic model.

Prob.: conditional probability of each topic to a drug.

Topic model ranks topics in accordance to their probability to each drug.

## Discussion

In this proof-of-concept study, topic modeling was demonstrated to be a promising approach to leverage information from drug safety databases comprised of textual data. As a case study, LiverTox database was explored by topic modeling to discover the hidden pattern for the identification of drugs potentially causing ALF. We deliberately chose to analyze the LiverTox "Hepatotoxicity" section alone so the findings could be verified by other sections in the LiverTox to demonstrate the potential utility of topic model in the field of drug safety. Specifically, first, ALF-Topic from the "Hepatotoxicity" sections of the drug documents was discovered, which was interpreted by the prevalence of three specific words (i.e., *jaundice*, *hepatocellular*, and *severe*). Then, this topic was applied to identify ALF-related drugs in the LiverTox database. Thereafter, evidence supporting risk of ALF for those identified drugs was found from the "Outcome and management" sections of the LiverTox or found from other public databases if not available from the LiverTox.

ALF-Topic was confirmed to be relevant to the well-known Hy's law [16,17], which defines that the observed drug-induced hepatocellular liver injury pattern together with jaundice has a poor prognosis with 10~50% fatality of ALF. The predictive power of Hy's law has been verified by the analysis of extensive studies in Spain and Sweden [18,19], and it has been recommended by the FDA for assessing the potential of a drug to cause severe DILI [20]. In this study, ALF-Topic identified 127 drugs in the LiverTox database, and approximately 60% (77/127) of these drugs were implicated to cause ALF in their "Outcome and management" sections. For those unspecified drugs, supporting evidence was found for 20 drugs in safety sections of their FDA-approved drug labels or in the literature with established ALF causality. ALF case reports were identified in the FAERS for the other 22 drugs, among which, 6 drugs were predicted as ALF positive drugs by an *in vitro *experiments. While the *in vitro *data might not directly indicate the ALF potential in humans, it was demonstrated that these 6 drugs were much closer to the ALF positive control drugs when they were tested by *in vitro *experiment. Evidence of ALF from the FAERS should be interpreted cautiously, because the causality may not be fully established. For example, although ALF cases of Phenelzine were reported in the FAERS, it was emphasized that Phenelzine might not be the suspect drug [21]. In addition to the overestimated risk, the FAERS only receives reports from the United States. For example, Ethionamide was not reported ALF in the FAERS despite being known to cause ALF in the United Kingdom [22].

For 127 identified drugs, evidence supporting risk of ALF was found for 119 drugs. The result strongly suggests that not only the specific wording but also their probabilistic/statistic relationship in the hidden structure of textual documents were crucial to incriminate drugs for ALF. It is worthwhile to point out that it is beyond the scope of this excise to ask ALF-Topic to identify all ALF-related drugs because ALF mechanisms are complex and the selected 26 known ALF drugs for determining ALF-Topic do not necessary represent the entire landscape of ALF. For example, hepatocellular liver injury pattern is not observed for Efavirenz and Dicloxacillin, which have the potential to cause ALF [12]. Atorvastatin and Ethambutol, known as ALF drugs [12], are not mentioned to cause either jaundice or hepatocellular liver injury in the LiverTox database.

## Conclusions

We explored the LiverTox database using topic modeling, and discovered the hidden knowledge to identify drugs with potential to cause ALF. Our proof-of-concept study demonstrates the applicability of topic modeling to leverage information within the textual drug safety databases, which will provide new opportunities for drug safety assessment.

## List of abbreviations used

ALF: Acute Liver Failure; DILI: Drug-Induced Liver Injury; FAERS: Food and Drug Administration (FDA) Adverse Event Reporting System; LDA: Latent Dirichlet Allocation; NIDDK: National Institute of Diabetes and Digestive and Kidney Diseases; NIH: National Institutes of Health; NLM: National Library of Medicine; MedDRA: Medical Dictionary for Regulatory Activities.

## Competing interests

The authors declare that they have no competing interests.

## Authors' contributions

WT and KI designed and supervised this study. KY and XX performed text mining experiments. KY, KI, MC, AS, and WT contributed to the writing of this manuscript. JZ performed the *in vitro *experiment.

## Supplementary Material

Additional file 1 **Table S1**. Summary of 77 ALF-implicated drugs specified to cause ALF in the LiverTox database. Click here for file

Additional file 2 **Table S2**. Number of ALF case reports by searching acute hepatic failure and/or hepatic failure in the FAERS for the 30 drugs implicated by the ALF-Topic. Click here for file

## Declarations

This study and publication was fully financed by the U.S. Food and Drug Administration (E0721511).

This article has been published as part of *BMC Bioinformatics *Volume 15 Supplement 17, 2014: Selected articles from the 2014 International Conference on Bioinformatics and Computational Biology. The full contents of the supplement are available online at http://www.biomedcentral.com/bmcbioinformatics/supplements/15/S17.
