# Supplementary material for: Mining hidden knowledge for drug safety assessment: topic modeling of LiverTox as a case study
Source: BMC Bioinformatics. 2014 Dec 16;15(Suppl 17):S6. doi: 10.1186/1471-2105-15-S17-S6 (PMC4304199; doi:10.1186/1471-2105-15-S17-S6)
Supplement: Additional file 1 — Table S1. Summary of 77 ALF-implicated drugs specified to cause ALF in the LiverTox database. [file 1471-2105-15-S17-S6-S1.doc]

**Table S1 Summary of 77 ALF-implicated drugs specified to cause ALF in the LiverTox database**

| **Drug name** | **1^st^ topic** | | **2^nd^ topic** | | **3^rd^ topic** | | **ALF-related terms in the LiverTox database** |
| --- | --- | --- | --- | --- | --- | --- | --- |
|  | **Topic ID** | **Prob.** | **Topic ID** | **Prob.** | **Topic ID** | **Prob.** |  |
| Allopurinol | 37 | 3.8E-01 | 7 | 3.1E-01 | 16 | 2.0E-01 | acute liver failure; fatal |
| AlphaInterferon and Peginterferon | 37 | 4.9E-01 | 39 | 2.3E-01 | 33 | 1.0E-01 | acute liver failure |
| Amitriptyline | 13 | 6.7E-01 | 37 | 1.9E-01 | 21 | 1.4E-01 | acute liver failure; fatal |
| Amodiaquine | 37 | 4.9E-01 | 16 | 2.4E-01 | 17 | 1.6E-01 | fatal; liver transplantation |
| Amphetamines | 37 | 6.4E-01 | 29 | 1.8E-01 | 36 | 1.1E-01 | acute liver failure; liver transplantation |
| Artemisinin derivatives | 37 | 7.9E-01 | 5 | 2.1E-01 | 40 | 4.7E-05 | fatal; liver transplantation |
| Atomoxetine | 37 | 1.0E+00 | 40 | 4.8E-05 | 39 | 4.8E-05 | acute liver failure; liver transplantation |
| Azathioprine | 37 | 6.2E-01 | 35 | 3.8E-01 | 40 | 7.3E-05 | hepatic failure |
| Benazepril | 21 | 6.9E-01 | 8 | 2.0E-01 | 37 | 1.0E-01 | acute liver failure |
| Black Cohosh | 37 | 8.5E-01 | 34 | 1.5E-01 | 40 | 3.1E-05 | acute hepatic failure; death |
| Carboplatin | 40 | 6.7E-01 | 37 | 3.1E-01 | 10 | 1.6E-02 | acute liver failure; death |
| Certolizumab | 37 | 4.5E-01 | 30 | 3.5E-01 | 39 | 1.7E-01 | hepatic failure; death |
| Chaparral | 37 | 7.6E-01 | 18 | 1.5E-01 | 29 | 9.2E-02 | acute liver failure |
| Clopidogrel | 28 | 8.0E-01 | 37 | 2.0E-01 | 40 | 3.9E-05 | acute liver failure; death |
| Clozapine | 19 | 5.7E-01 | 37 | 4.3E-01 | 40 | 3.3E-05 | acute liver failure |
| Comfrey | 37 | 5.4E-01 | 40 | 2.7E-01 | 27 | 1.9E-01 | acute liver failure |
| Dacarbazine | 40 | 6.1E-01 | 37 | 3.4E-01 | 11 | 4.4E-02 | fatal hepatic injury |
| Dantrolene | 37 | 1.0E+00 | 40 | 3.9E-05 | 39 | 3.9E-05 | acute liver failure; death |
| Delavirdine | 37 | 4.7E-01 | 7 | 2.1E-01 | 12 | 1.8E-01 | acute liver failure; liver transplantation |
| Didanosine | 37 | 8.8E-01 | 39 | 1.2E-01 | 40 | 5.8E-05 | hepatic failure |
| Emtricitabine | 26 | 6.3E-01 | 21 | 1.3E-01 | 37 | 1.2E-01 | acute liver failure; liver transplantation |
| Ephedra | 37 | 6.5E-01 | 27 | 1.5E-01 | 29 | 9.9E-02 | acute liver failure |
| Erythromycin | 28 | 3.8E-01 | 17 | 3.0E-01 | 37 | 1.6E-01 | acute liver failure; death; transplantation |
| Etanercept | 37 | 3.4E-01 | 28 | 2.6E-01 | 39 | 2.2E-01 | hepatic failure; death |
| Ethionamide | 37 | 5.1E-01 | 5 | 4.6E-01 | 10 | 3.0E-02 | fatal |
| Etodolac | 38 | 5.5E-01 | 37 | 3.9E-01 | 36 | 4.8E-02 | fatal |
| Etravirine | 5 | 4.6E-01 | 12 | 2.9E-01 | 37 | 1.9E-01 | acute liver failure |
| Felbamate | 37 | 6.1E-01 | 35 | 3.9E-01 | 40 | 4.9E-05 | acute liver failure |
| First generation sulfonylureas | 37 | 6.1E-01 | 10 | 3.9E-01 | 40 | 2.3E-05 | hepatic failure; death |
| Flutamide | 37 | 5.1E-01 | 2 | 2.2E-01 | 31 | 1.8E-01 | acute liver failure; liver transplantation |
| Germander | 37 | 5.8E-01 | 27 | 4.0E-01 | 3 | 1.9E-02 | acute liver failure; liver transplantation |
| Green tea | 37 | 5.0E-01 | 27 | 4.7E-01 | 4 | 2.9E-02 | liver transplantation |
| Ifosfamide | 40 | 6.9E-01 | 19 | 1.6E-01 | 37 | 1.2E-01 | acute liver failure; fatal hepatic necrosis |
| Imipramine | 13 | 7.4E-01 | 37 | 2.6E-01 | 40 | 3.8E-05 | acute liver failure; death |
| Indomethacin | 37 | 6.3E-01 | 31 | 2.3E-01 | 39 | 9.5E-02 | acute liver failure; death |
| Itraconazole | 5 | 6.0E-01 | 37 | 4.0E-01 | 40 | 4.2E-05 | acute liver failure; death; transplantation |
| Kava Kava | 37 | 4.8E-01 | 36 | 2.5E-01 | 9 | 2.0E-01 | acute liver failure; fulminant hepatitis |
| Ketoconazole | 37 | 7.6E-01 | 3 | 2.4E-01 | 40 | 4.3E-05 | acute liver failure; liver transplantation |
| Labetalol | 5 | 4.5E-01 | 37 | 3.3E-01 | 32 | 2.1E-01 | acute liver failure; liver transplantation |
| Lamivudine | 26 | 7.0E-01 | 37 | 3.0E-01 | 40 | 2.5E-05 | liver failure; liver transplantation |
| Lamotrigine | 7 | 3.9E-01 | 37 | 3.2E-01 | 9 | 1.6E-01 | acute liver failure |
| Lisinopril | 21 | 5.7E-01 | 37 | 2.6E-01 | 8 | 1.7E-01 | acute liver failure; death |
| Melphalan | 40 | 6.7E-01 | 37 | 3.3E-01 | 39 | 2.3E-05 | acute liver failure; fatal |
| Mercaptopurine | 37 | 5.2E-01 | 18 | 4.8E-01 | 40 | 8.6E-05 | hepatic failure |
| **Drug name** | **1^st^ topic** | | **2^nd^ topic** | | **3^rd^ topic** | | **ALF-related terms in LiverTox database** |
|  | **Topic ID** | **Prob.** | **Topic ID** | **Prob.** | **Topic ID** | **Prob.** |  |
| Metronidazole | 37 | 6.9E-01 | 20 | 2.3E-01 | 17 | 6.4E-02 | liver failure; death |
| Moxifloxacin | 15 | 5.3E-01 | 37 | 2.2E-01 | 14 | 1.7E-01 | liver failure; fulminant |
| Nevirapine | 37 | 5.8E-01 | 16 | 1.7E-01 | 12 | 1.3E-01 | fatal; liver transplantation; acute liver failure |
| Niacin | 37 | 6.5E-01 | 20 | 1.8E-01 | 2 | 1.6E-01 | liver failure; fatal; liver transplantation |
| Nilutamide | 13 | 3.5E-01 | 37 | 3.3E-01 | 36 | 1.7E-01 | fatal |
| Noni | 37 | 1.0E+00 | 40 | 6.6E-05 | 39 | 6.6E-05 | acute liver failure; liver transplantation |
| Nortriptyline | 13 | 8.1E-01 | 37 | 1.9E-01 | 40 | 4.7E-05 | acute liver failure |
| Oxaprozin | 37 | 5.3E-01 | 38 | 3.7E-01 | 7 | 9.2E-02 | acute liver failure |
| Paroxetine | 19 | 8.3E-01 | 37 | 1.7E-01 | 40 | 5.3E-05 | acute liver failure |
| Penicillin G and V | 37 | 4.1E-01 | 4 | 2.3E-01 | 7 | 1.7E-01 | fatal |
| Pennyroyal oil | 37 | 5.5E-01 | 29 | 3.5E-01 | 19 | 9.8E-02 | acute liver failure; death |
| Piroxicam | 37 | 1.0E+00 | 40 | 4.7E-05 | 39 | 4.7E-05 | acute liver failure |
| Propylthiouracil | 37 | 9.9E-01 | 38 | 1.1E-02 | 40 | 2.8E-05 | hepatic failure; death; liver transplantation |
| Pyrimethamine/Sulfadoxine | 7 | 6.8E-01 | 37 | 2.4E-01 | 14 | 7.6E-02 | acute liver failure |
| Second generation sulfonylureas | 37 | 6.9E-01 | 10 | 3.1E-01 | 40 | 3.0E-05 | hepatic failure; death |
| Sertraline | 19 | 9.3E-01 | 37 | 6.5E-02 | 40 | 5.4E-05 | acute liver failure |
| Stavudine | 37 | 4.9E-01 | 6 | 2.4E-01 | 39 | 2.0E-01 | acute liver failure; death |
| Sulfadiazine | 7 | 7.5E-01 | 37 | 2.4E-01 | 40 | 2.5E-05 | acute liver failure; acute hepatic failure |
| Sulindac | 37 | 5.5E-01 | 15 | 2.5E-01 | 7 | 1.5E-01 | acute liver failure; death |
| Sunitinib | 31 | 4.6E-01 | 2 | 3.4E-01 | 37 | 1.9E-01 | acute liver failure |
| Tamoxifen | 5 | 3.7E-01 | 37 | 3.3E-01 | 35 | 2.7E-01 | hepatic failure |
| Tenofovir | 26 | 6.4E-01 | 37 | 2.3E-01 | 30 | 1.2E-01 | acute liver failure; liver transplantation |
| Telithromycin | 28 | 5.4E-01 | 37 | 2.6E-01 | 17 | 2.0E-01 | liver failure; liver transplantation |
| Terbinafine | 37 | 7.9E-01 | 16 | 9.7E-02 | 21 | 7.3E-02 | acute liver failure; liver transplantation |
| Tetracycline/Oxytetracyline | 37 | 4.2E-01 | 6 | 3.2E-01 | 22 | 1.4E-01 | fatality; liver failure; death |
| Thioguanine | 37 | 6.5E-01 | 35 | 3.5E-01 | 40 | 5.8E-05 | hepatic failure |
| Thiotepa | 40 | 5.9E-01 | 37 | 3.9E-01 | 28 | 2.1E-02 | hepatic failure; acute liver failure; fatalities |
| Tramadol | 29 | 4.6E-01 | 14 | 2.5E-01 | 37 | 1.5E-01 | acute liver failure; fatal |
| Usnic Acid | 37 | 6.0E-01 | 27 | 3.5E-01 | 18 | 5.0E-02 | acute liver failure; liver transplantation |
| Warfarin | 37 | 7.5E-01 | 17 | 2.2E-01 | 15 | 3.3E-02 | hepatic failure; death |
| Zafirlukast | 37 | 5.6E-01 | 31 | 2.3E-01 | 20 | 1.3E-01 | fulminant hepatitis; hepatic failure |
| Zidovudine | 37 | 5.5E-01 | 6 | 2.1E-01 | 39 | 1.6E-01 | hepatic failure; death |
| Zileuton | 31 | 5.2E-01 | 37 | 2.3E-01 | 28 | 2.3E-01 | hepatic failure |
| Topic-37 is the ALF-Topic.  Topic ID: serial number of topics in topic model.  Prob.: conditional probability of each topic to a drug.  Topic model ranks topics in accordance to their probability to each drug. | | | | | | | |
