# Supplementary material for: Mining hidden knowledge for drug safety assessment: topic modeling of LiverTox as a case study
Source: BMC Bioinformatics. 2014 Dec 16;15(Suppl 17):S6. doi: 10.1186/1471-2105-15-S17-S6 (PMC4304199; doi:10.1186/1471-2105-15-S17-S6)
Supplement: Additional file 2 — Table S2. Number of ALF case reports by searching acute hepatic failure and/or hepatic failure in the FAERS for the 30 drugs implicated by the ALF-Topic. [file 1471-2105-15-S17-S6-S2.doc]

**Table S2 Number of ALF case reports by searching *acute hepatic failure* and/or *hepatic failure*** in the FAERS for the 30 drugs implicated by the ALF-Topic

| **Drug name** | **Number of ALF case reports** | **Drug name** | **Number of ALF case reports** |
| --- | --- | --- | --- |
| Metformin | 139 | Acebutolol | 3 |
| Olanzapine | 137 | Phenelzine | 2 |
| Adefovir | 69 | Mebendazole | 2 |
| Linezolid | 61 | Thioridazine | 2 |
| Ceftriaxone | 60 | Ticarcillin/clavulanate | 2 |
| Risperidone | 52 | Trisalicylate | 1 |
| Fenofibrate | 45 | Orphenadrine | 1 |
| Montelukast | 33 | Clofibrate | 0 |
| Verapamil | 22 | Reserpine | 0 |
| Sotalol | 10 | Methocarbamol | 0 |
| Colchicine | 6 | Pentamidine | 0 |
| Oxacillin | 5 | Aloe Vera^*^ | 0 |
| Nifedipine | 4 | Ba Jiao Lian^*^ | 0 |
| Alfuzosin | 4 | Chi R Yun^*^ | 0 |
| Gold salts | 3 | Shosaikoto/daisaikoto^*^ | 0 |
| ^*^Herbal medicines are not recorded in the FAERS.  ALF: acute liver failure.  FAERS: FDA Adverse Event Reporting System. | | | |
